# No Evidence of On-farm Circulation of Avian Influenza H5 Subtype in Ca Mau Province, Southern Vietnam, March 2016 – January 2017

**DOI:** 10.1371/currents.outbreaks.c816d7333370d68f8a0da33f69168986

**Published:** 2017-05-05

**Authors:** Nguyen Thi Le Thanh, Nguyen Ha Thao Vy, Huynh Thi Ai Xuyen, Huynh Thi Phuong, Phung Ngoc Tuyet, Nguyen Thanh Huy, Benjamin Nguyen-Van-Yen, Ha Minh Lam, Maciej F Boni

**Affiliations:** Oxford University Clinical Research Unit, Wellcome Trust Major Overseas Programme, Ho Chi Minh City, Vietnam; Oxford University Clinical Research Unit, Wellcome Trust Major Overseas Programme, Ho Chi Minh City, Vietnam; Ca Mau sub-Department of Livestock Prodution and Animal Health, Ward 5, Ca Mau City, Ca Mau, Vietnam; Oxford University Clinical Research Unit, Wellcome Trust Major Overseas Programme, Ho Chi Minh City, Vietnam; Ca Mau sub-Department of Livestock Prodution and Animal Health, Ward 5, Ca Mau City, Ca Mau, Vietnam; Ca Mau sub-Department of Livestock Prodution and Animal Health, Ward 5, Ca Mau City, Ca Mau, Vietnam; Oxford University Clinical Research Unit, Wellcome Trust Major Overseas Programme, Ho Chi Minh City, Vietnam; Department of Biology, École Normale Supérieure, Paris, France; Oxford University Clinical Research Unit, Wellcome Trust Major Overseas Programme, Ho Chi Minh City, Vietnam; Department of Biology, Pennsylvania State University, University Park, PA, USA; Oxford University Clinical Research Unit, Wellcome Trust Major Overseas Programme, Ho Chi Minh City, Vietnam

## Abstract

**Background:**

Subtype H5N1 avian influenza viruses, both high pathogenicity and low pathogenicity, have been enzootic in Vietnam since 2001.  The viruses are readily identified at live bird markets, but virus prevalence on smallholder poultry is typically zero or very low.  If the true direction of the viral transmission chain is farm to market, it is unknown why farm prevalence should be low when market prevalence is moderate to high.

**Methods:**

We established a cohort of 50 smallholder poultry farms in Ca Mau province in the Mekong Delta region of Vietnam.  From March 2016 to January 2017, we collected naso-pharyngeal and cloacal samples from 156 ducks and 96 chickens.  In addition, 126 environmental samples were collected.  Samples were assayed for H5 subtype influenza by real-time RT-PCR.

Results/Discussion: None of the 378 collected samples were positive for H5 influenza.  This is likely to mean that circulation of subtype H5 influenza viruses was low in Ca Mau in 2016.  Detection of avian influenza on smallholder poultry farms is necessary to determine the directionality and association between farm prevalence and market prevalence of avian influenza viruses.  Larger farm-level studies should be planned as these will be critical for determining the presence and strength of this association.

## Introduction

Avian influenza viruses are endemic in poultry populations in South East Asia. The prevalence of the virus varies substantially by region. Outbreaks can be reported at any time, although they are more commonly reported during new year or other festival celebrations. Outbreaks of disease can occur both in markets and on farms^1^. In healthy birds however, viral prevalence is occasionally detected at live poultry markets^2^^,^^3^^,^^4^ but rarely on farms^4^^,^^5^^,^^6^. This is unexpected because poultry spend the first 6 to 8 weeks of their life on a farm and typically only the last 6-12 hours at a poultry market^20^^,^^21^. The natural directionality of transmission that one would expect is that viral positivity in markets is seeded by viral positivity on farms, but this chain of infection is difficult to capture with a convincing set of data points.

The Mekong Delta region of Vietnam is an epicenter of avian influenza circulation, with regular outbreaks 1 of avian influenza on smallholder farms and scores of human cases occurring since 2004 (127 in Vietnam as a whole^7^). In 2015, avian influenza prevalence was reported from market surveys of healthy birds in Ca Mau province, the southernmost province in the Mekong Delta region, approximately 240km southwest of Ho Chi Minh City. After routine sampling at rural live bird markets (LBM) by the Ca Mau sub-Department of Livestock Production and Animal Health (CM-LPAH), molecular diagnostics on pooled samples indicated that prevalence of the H5 influenza subtype in individual birds is likely between 4.2% and 6.3%. Molecular diagnostics performed on environmental samples (feces, feathers) from the same markets pointed to an individual-bird H5 prevalence between 1.4% and 4.5% (internal reports, CM-LPAH). These percentages were slightly higher for 2016 (internal reports, CM-LPAH). Subtype H5N1 avian influenza viruses have been circulating in Ca Mau province since 2005 or earlier^8^^,^^9^^,^^10^ with outbreaks reported regularly between 2005 and 2016^1^^,^^11^^,^^12^, and for these reasons Ca Mau province was thought to be a potential reservoir region where avian influenza would be able to circulate and persist on smallholder farms.

To aid the CM-LPAH in further investigating these results, we established a cohort of 50 smallholder poultry farms, carried out on-farm monthly poultry censuses, and performed molecular diagnostics for subtype H5 avian influenza in healthy birds sampled from March 2016 to January 2017.

## Methods

With the help of the Ca Mau sub-Department of Livestock Production and Animal Health, 50 smallholder poultry farms were identified in Tan Loc commune and Tan Phu commune (25 in each; see Figure 1) and enrolled in the study. These communes were chosen by CM-LPAH for their past history of avian influenza outbreaks, high farm density, expected participation rate, and proximity to the main city in the province. The inclusion criteria were designed so that the cohort would include both large and small farms as well as majority chicken and majority duck farms. Farms were defined as small if they had between 20 and 100 birds, and large if they held over 100 birds. For chicken farms, we aimed to enroll 80% small farms and 20% large farms; for duck farms, the aim was 50% large and 50% small. The farms had a range of species compositions, and the species compositions changed through time. Other birds raised on these farms included Muscovy ducks, geese, quail, turkeys, and pigeons. The study was designed to generate a time series of poultry population sizes together with a time series of avian influenza positivity, in order to determine if the two variables were correlated in time. Monthly poultry counts – which included basic information on species, age, sales, purchases, and deaths – began on all 50 farms in June 2015. A pilot study collecting cloacal and naso-pharyngeal swabs from individual birds, as well as fecal and feather samples collected from the environment, was initiated in March 2016. The research collaboration was approved by the Hospital for Tropical Diseases in Ho Chi Minh City, and the study design was approved by the Ca Mau sub-Department of Livestock Production and Animal Health. The Ca Mau sub-Department of Livestock Production and Animal Health (CM-LPAH) specifically approved this study and is equivalent to an Animal Care and Use Committee that approves studies like this.

The four sample collection periods chosen were March (3 farms), August (6 farms), November (6 farms) 2016, and January 2017 (6 farms). The farms were chosen by selecting those that had a sufficient population size for sampling, birds that were at least six weeks old, and presence of chickens and ducks across all the selected farms so that chickens and ducks could be sampled in approximately equal numbers. Eighteen samples were collected on each farm: six naso-pharyngeal (NP) samples, six cloacal (CL) samples, three fecal samples, and three feather samples. Each NP and CL sample was a pool of two swabs from two different birds. The NP and CL samples were matched, i.e. collected from the same birds. Swabs and feathers were placed in viral transport medium, and all samples were transported to Ho Chi Minh City on dry ice (by car, ~6 hours) and stored at -80°C at the Oxford University Clinical Research Unit. A total of 378 samples were collected over the 10-month period of sampling. Assuming a per-bird influenza prevalence of 2.0%, the study would yield five or more positive samples with a probability of 87.5%. The sampling design and sampling numbers were based on the 2015 CM-LPAH routine surveillance reports.

**Map of study area in southern Vietnam.**
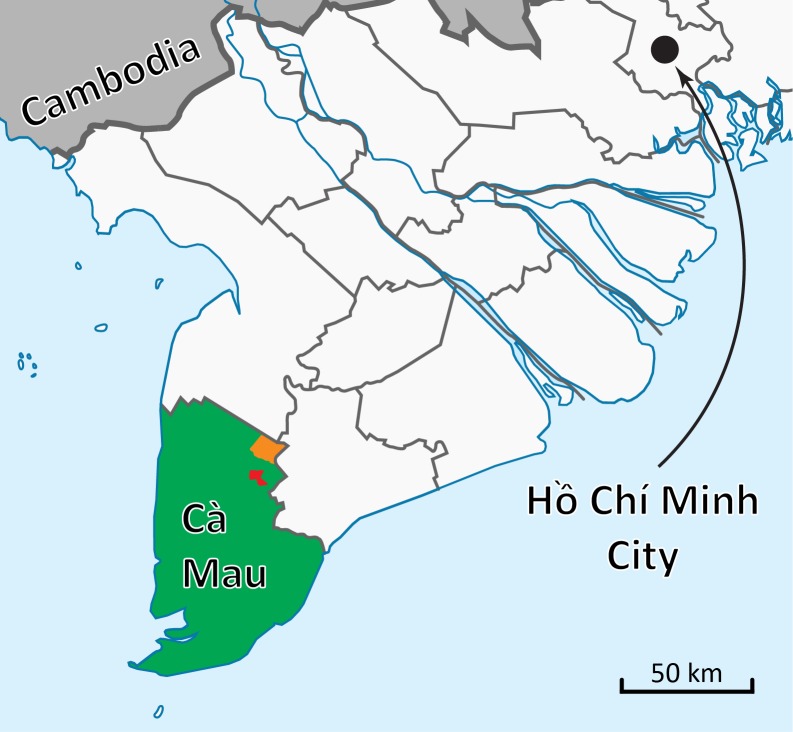
Ca Mau province is shown in green. Tan Loc commune (red) is about 10km from Ca Mau city (centrally located in the province). Tan Phu commune (orange) is about 20km from Ca Mau City.

**Table 1:** Primer and probe sets used in real-time RT-PCR assay.

Primers and Probes	Sequence (5’ > 3’)
As H5a Forward	TGG AAA GTG TAA RAA ACG GAA CGT
As H5a Reverse	YGC TAG GGA ACT CGC CAC TG
As H5a Probe1	FAM-TGA CTA CCC GCA G”T”A TTC AGA AGA AGC AAG ACT AA-BHQ1
As H5a Probe2	FAM-CAA CTA TCC GCA G”T”A TTC AGA AGA AGC AAG ATT AA-BHQ1
As H5b Forward	GGA ATG CCC CAA ATA TGT GAA ATC AA
As H5b Reverse	CTC CCC TGC TCA TTG CTA TGG T
As H5b Probe	Cyan500-TAC CCA TAC CAA CCA “T”CT ACC ATT CCC TGC CAT-BBQ

Viral RNA was extracted from 140uL of each pooled specimen to obtain a final eluted volume of 60uL. The procedures were carried out manually by using the commercial QIAamp Viral RNA Mini kit (Qiagen, Cat ID.52906), and the viral extract was stored at -80°C for further testing. All extracted specimens were PCR-screened for the presence of the H5 influenza subtype influenza virus by real-time RT-PCR. Primer and probe sets recommended by Centers for Disease Control and Prevention^13^ were used, and are identical to those used in previous avian influenza studies in southern Vietnam^11^^,^^14^. Primers and probes are listed in Table 1; both H5a and H5b primers were used.

PCR amplification was performed using a LightCycler 480 RNA Master Hydrolysis Probes (Roche; CatID. 04991885001) and performed on a LightCycler® 480 Instrument II (Roche). Each reaction had a total volume of 20uL containing 5uL of the viral RNA extract, 1X of RNA Master Hydrolysis Probes, 3.25mM of Mn(OAc)2, 1X of enhancer solution, 0.2uM of avian influenza H5a/H5b probes, 0.8uM avian Influenza H5a/H5b primer (each), and water.

No template controls (NTC) and viral template controls (VTC) for all primer and probe sets were included in each run as quality control reactions. Thermal cycling conditions were as follows: reverse transcription at 58°C for 20 minutes, enzyme inactivation at 95°C for 5 minutes, and 45 cycles of 95°C for 15 seconds, 55°C for 30 seconds, and 72°C for 20 seconds. Data were analyzed using LightCycler 480 software. A sample was considered positive if its Cp value was ≤ 40. Positive and negative controls showed presence and absence of amplification, respectively.

## Results

Fifty farms were enrolled in June 2015, and the first 6-8 months of the study were run as a pilot to determine if data collection (poultry counts) was simple, reliable, and sustainable. The farms had changing species compositions and changing population sizes throughout the course of the study. Although the original intent of the study was to identify “majority chicken” and “majority duck” farms, about half of the farms had as assortment of chickens, ducks, and Muscovy ducks, and they could not be classified as primarily a single species farm.

A total of 21 smallholder farms were chosen for poultry sampling between March 2016 and January 2017 (Table 2), with farm sizes ranging from 29 to 572 birds; the only farm outside this range had 4700 quails, 285 ducks, and 60 chickens. Naso-pharyngeal swabs and cloacal swabs were taken from 156 ducks and 96 chickens; all birds were six weeks or older in age. Environmental sampling included 47 duck feces samples, 47 duck feather samples, 16 chicken feces samples, and 16 chicken feather samples. All 378 samples tested negative for H5 influenza by real-time PCR, resulting in an estimated H5 prevalence of 0.0% (95% CI: 0.0% – 0.97%). The simplest hypothesis explaining this result is that circulation of the H5 subtype in Ca Mau province was low in 2016.

All sampled birds were healthy at time of sampling. One sampled farm reported 44% disease-induced poultry mortality during the month of sampling (August 2016), and as there were too few chickens on this farm sampling was continued on a separate farm to obtain the full complement of 18 samples. The remaining 20 farms reported lower than 10% mortality in adult poultry during the month of sampling, which is within the normal range of mortality for smallholder poultry in Vietnam.

**Table 2:** Numbers of samples taken and numbers of birds present on each farm at the beginning of the month of sampling. All samples were negative for subtype H5 influenza. ^a,b^sampling on farm 6 was carried out on two farms (a and b) as 16/20 chickens had died on farm a, and it was decided to carry out chicken sampling on a new farm (farm b).

Farm ID	Month	Ducks Sampled / Ducks Present	Chickens Sampled / Chickens Present	Other Birds Present	Feces/Feather Samples Taken
1	Mar 2016	6 / 510	6 / 36	19	6
2	Mar 2016	6 / 15	6 / 170	0	6
3	Mar 2016	12 / 495	0 / 0	0	6
4	Aug 2016	6 / 200	6 / 75	15	6
5	Aug 2016	6 / 25	6 / 113	9	6
6	Aug 2016	6 / 30^a^	6 / 120^b^	0^a^, 53^b^	6
7	Aug 2016	6 / 6	6 / 566	0	6
8	Aug 2016	6 / 285	6 / 60	4700	6
9	Aug 2016	12 / 70	0 / 8	7	6
10	Nov 2016	6 / 25	6 / 22	0	6
11	Nov 2016	6 / 45	6 / 17	0	6
12	Nov 2016	12 / 58	0 / 0	30	6
13	Nov 2016	6 / 47	6 / 215	0	6
14	Nov 2016	6 / 28	6 / 25	10	6
15	Nov 2016	6 / 120	6 / 110	58	6
16	Jan 2017	12 / 15	0 / 0	14	6
17	Jan 2017	6 / 31	6 / 6	0	6
18	Jan 2017	12 / 18	0 / 0	17	6
19	Jan 2017	6 / 119	6 / 45	2	6
20	Jan 2017	6 / 25	6 / 192	6	6
21	Jan 2017	6 / 87	6 / 262	0	6

## Discussion

The vast majority of Vietnam’s poultry sector is still smallholder poultry, with more than 96% of poultry-owning households holding fewer than 100 chickens^15^ and more than 93% of poultry-owning households holding fewer than 100 ducks^16^. Outbreaks of avian influenza on poultry farms are common and have been recorded every year in Vietnam^1^^,^^17^ since the re-emergence in 2002 and 2003 of highly-pathogenic avian influenza H5N1 genotypes in Asia^18^^,^^19^. Live poultry sales in Vietnam take place in a large informal system of household sales and wet markets, with some markets occupying the place of a central town/city market and others emerging temporarily on roadsides or in the countryside. Avian influenza viruses are detectable in markets, both in healthy and diseased birds, but contemporaneous sampling of healthy birds in markets and farms rarely shows any positive results on farms^4^. There is as yet no evidence that the virus can persist in market areas, and thus the more frequent direction of viral transmission is likely to be from farm to market. However, the informal nature of the sales, transportation, and storage chains has not been studied in enough detail to state with certainty that the virus cannot persist in market environments.

The logical mechanism of farm-to-market transmission would enjoy more evidential support if avian influenza viruses could be detected on farms at prevalence levels close to those at which they are observed in markets. A study aimed at gathering this evidence would require a long-term cohort of farms in close proximity to market areas, so that farm prevalence could be investigated when or if avian influenza is detected in markets, either through active or passive surveillance. However, the necessary sample size – both number of farms and number of individual poultry – is likely to be large as expected avian influenza prevalence in healthy poultry is below 1%, with a higher chance of finding avian influenza in healthy ducks than in healthy chickens or healthy Muscovy ducks. For this simple reason, this type of study is difficult to implement and carries with it a substantial chance of failure as sufficient numbers of positive birds may not be identified on farms during periods when market outbreaks are occurring. Nevertheless, it is crucial that research communities focused on animal health and zoonotic pathogens begin designing these types of large studies^22^ so that we can come to stronger conclusions on the origins of avian influenza viruses detected at live poultry markets. In our study, the number of H5N1 positive birds from 21 farms was zero during a 10-month period, despite all sampled birds being six weeks or older; this prevalence was lower than our expectation at the beginning of the study. This means that data points covering the hypothesized farm-to-market transmission chain will be difficult to assemble without a geographically broad cohort of approximately >100 farms and >1000 samples taken per year. The opinion of veterinary staff working at CM-LPAH is that market prevalence is higher than farm prevalence due to (1) many poultry at markets in Ca Mau being imported from other provinces, (2) a lack of hygiene and disinfection at markets compared to the hygiene practiced by individual smallholder farmers, and (3) the enrolled farmers in this specific study receiving information on disease prevention practices and being aware that they would be interacting with the CM-LPAH once a month.

A similar pattern of market positivity and farm negativity has been observed in the H7N9 outbreaks in China. The initial wave of infections in 2013 affected urban residents more than rural residents^23^. Poultry samples collected during this time were H7N9-positive only if collected at live bird markets, and samples collected from poultry farms were negative^24^. In addition, closing LBMs had the expected effect of interrupting zoonotic transmission from poultry to humans^25^. Documented evidence of H7N9 infection risk on poultry farms does exist^2^^,^^27^^,^^28^, but the majority of human infections have been reported from LBMs in urban areas. Although it is ‘common knowledge’ that H7N9 viruses are not detected on poultry farms^29^, negative results are rarely published, and we cannot determine if this lack of viral detection is caused by a lack of farm level studies or unpublished negative results.

In addition to larger sample size, a second study design feature that would be helpful in establishing the chain of transmission would be farm-centered questionnaires (as in^30^^,^^31^ ) that track sales locations (almost all poultry from smallholder farms are eventually sold at markets, and a small proportion are slaughtered on-site for personal consumption). The usefulness of this type of information can be seen in the market-network analyses of Fournié et al^20^^,^^21^ which show that poultry at live bird markets can come from a variety of sources including other LBMs. At small LBMs farmers are likely to sell their own poultry while at larger ones, sellers are likely to be selling poultry aggregated through a trading network^21^. With a farm-centered analysis, we would be able to determine if smallholder farms send their finished poultry primarily to traders, nearby LBMs, or distant LBMs. This would give us a baseline expectation for whether viruses present at markets should be seeded by local circulation on poultry farms.

The primers and probes used in the molecular diagnostics in our study were designed more than ten years ago. They were successfully used to identify positive H5 viruses from poultry samples in outbreaks occurring in 2010^11^ and 2013^14^ in southern Vietnam, but we cannot exclude the possibility that mutations in the haemagglutinin gene of the H5N1 virus have caused this assay to have low sensitivity. Outbreaks reported in 2015 and 2016 – including outbreaks in Ca Mau in both years – were detected as positive by real-time RT-PCR, by the same Vietnamese government network of animal health laboratories that includes CM-LPAH. To our knowledge, the primers and probes used in these regional and provincial laboratories have not changed in the last few years.

The original study design aimed to generate a time series of avian influenza prevalence and poultry population sizes on a cohort of smallholder poultry farms. Our goal was to investigate changing population sizes on farms and the overlap among different flocks on the same farm, to determine if either of these could be linked to the persistence or prevalence of avian influenza virus on individual farms. Although it is clear that avian influenza is always present in some parts of southern Vietnam, it is not clear if the local presence/absence of the virus is driven primarily by season, poultry density, importation from wild birds, farming practice, or another dynamic causing the viral metapopulation to move into and out of different regions at different times. To begin understanding the regional circulation of the virus, broader and more regular sampling needs to be performed in markets and on farms, and crucially, it must become standard to publish negative results so that the positive results in the literature can be interpreted properly.

## Competing Interests

The authors have declared that no competing interests exist.

## Data Availability Statement

All data relevant to the conclusions presented in this article are included in the article text. Further details on these data can be obtained by emailing the authors.

## Funding Statement

The study was funded by the Defense Threats Reduction Agency (US) and by the Wellcome Trust grant (098511/Z/12/Z). The funders had no role in study design, data collection and analysis, decision to publish, or preparation of the manuscript.

## Corresponding Author

Corresponding Author Maciej F Boni, mfb9@psu.edu, Center for Infectious Disease Dynamics, Department of Biology, Pennsylvania State University
